# Acute Pyelonephritis by Scrub Typhus: A Rare Condition

**DOI:** 10.7759/cureus.55752

**Published:** 2024-03-07

**Authors:** Debashis Priyadarshan Sahoo, Soumya Ranjan Pradhan, Gwenette War, Annu Gupta, Ruth K Tara

**Affiliations:** 1 General Medicine, All India Institute of Medical Sciences, Guwahati, IND; 2 Radiology, Apollo Hospitals, Bhubaneswar, IND

**Keywords:** scrub typhus, doxycycline, septic shock, acute pyelonephritis, urinary tract infection

## Abstract

Scrub typhus is prevalent in tropical countries and can have a varied spectrum of presentations from pneumonia, gastroenteritis, lymphadenitis, meningitis, encephalitis, and acute kidney injury to multi-organ dysfunction syndrome. Urinary tract infections like cystitis and pyelonephritis are rarely reported.

Here we present an atypical presentation of a 53-year-old female with diabetes mellitus who came to the outpatient department with complaints of high-grade fever, burning micturition, and left flank pain for three days and was initially treated outpatient basis with oral antibiotics. However, her deteriorating condition landed her in an emergency in a state of septic shock. She was initially treated with broad-spectrum conventional antibiotics with other supportive medications. Even after confirmation of the diagnosis of left acute pyelonephritis with septic shock, with appropriate antibiotics, her condition was deteriorating. A sterile urine culture raised suspicion of atypical organisms causing the infection. Proper analysis of her history and readily available investigations of IgM against scrub typhus antigen led to a diagnosis of scrub typhus-related left acute pyelonephritis with septic shock. She was treated adequately with an injection of doxycycline, followed by oral tablets of the same, after which she showed drastic improvement in her symptoms, and then she was discharged.

Thus, atypical organisms causing urinary tract infections should be kept always in mind, which can be treated easily and if untreated, can lead to life-threatening consequences.

## Introduction

Scrub typhus, also known as tsutsugamushi disease, is a rickettsial illness caused by *Orientia tsutsugamushi*. This vector-born disease is transmitted to humans through the bites of infected chiggers (larval mites). It is endemic in rural areas of Asia, the western Pacific islands, and northern Australia [1]. Early diagnosis and prompt treatment with antibiotics are crucial for managing scrub typhus. The disease is characterized by fever, headache, muscle pain, and a distinctive eschar at the site of the chigger bite. This disease has variable presentations in the form of pneumonia, meningitis, encephalitis, lymphadenitis, gastroenteritis hepatitis, acute kidney injury (AKI), and multi-organ dysfunction. Due to its high morbidity and mortality, scrub typhus infection is considered to be serious and life-threatening; the case fatality rate can reach as high as 70% if untreated (median being 6%) [2]. Scrub typhus mortality is influenced by the virulence of the strains, patient characteristics, delay in diagnosis, and drug resistance.

Urinary tract infections, such as cystitis and pyelonephritis, are mainly caused by *Escherichia coli, Klebsiella, *and* Staphylococcus* and rarely by *Proteus* and *Ureaplasma* [3]. *Orientia tsutsugamushi* is a rare causative organism of urinary tract infections. Patients presenting with symptoms of urinary tract infections require a high degree of clinical suspicion for diagnosing scrub typhus which can be confirmed by polymerase chain reaction and indirect immunofluorescence assay [4]. A four-fold rise in IgM antibody titer is usually diagnostic of infection [4].

## Case presentation

A 53-year-old female with type 2 diabetes mellitus presented to the medicine outpatient department with complaints of intermittent high-grade fever, burning micturition, and left flank pain for three days. She had no similar history in the past and no such family history. She was married for 22 years with two living children and she was sexually inactive. She has been taking Tablet Glimepiride 1mg one tablet once daily for the last 11 years. During examination, she was afebrile, her blood pressure was 110/62 mmHg, pulse rate: 98/minute, and respiratory rate: 20/minute (abdominothoracic). She had no pallor, icterus, cyanosis, lymphadenopathy, or rashes and systemic examination revealed left renal angle tenderness without any significant respiratory, cardiovascular, and neurological abnormalities. 

On blood routine examination, hemoglobin, total leukocyte count (TLC), erythrocyte sedimentation rate (ESR), random blood sugar (RBS), serum urea, and creatinine were within normal limits (Table 1). Her urine analysis showed 1-2 pus cells/high power field without any red blood cells and casts (Table 2). Ultrasonography of the abdomen revealed features of urinary derbies and fatty liver grade I (Table 3). Urine culture and blood cultures were sent (Day 1). Tablet Cefixime 200mg twice daily for seven days and tab. Hyoscine 20mg (if pain) were advised with continuation of tablet Glimepiride 1mg once daily and she was asked to follow up with culture and sensitivity reports. 

**Table 1:** Serial monitoring of hematological and biochemical parameters. Hb: Hemoglobin; TLC: total leukocyte count

Parameters	Day 1	Day 3	Day 4	Day 7	Day 9	Day 10	Day 12	Reference range
Hb (%)	12.1	11.0	-	10.8	-	10.8	11.0	12-15
TLC (x 1000)	5.4	12.3	-	9.9	-	7.3	6.8	4-10
Platelet Count (x 1000)	265	256	-	254	-	266	276	150-410
Serum Urea (mg/dL)	22	156	211	98	-	21	18	14.98-36.38
Creatinine (mg/dL)	0.9	2.1	2.8	1.7	-	0.9	0.8	0.52-1.04
Albumin (mg/dL)	4.6	4.5		-	-	4.1	-	3.5 - 5
RBS (mg/dL)	188	178		-	-	165	166	< 200
Procalcitonin (ng/mL)	-	-	98		67	10	<0.5	< 0.05

**Table 2:** Urine analysis during outpatient visit and hospitalization. Hpf: High power field, RBC: Red blood cells.

Parameters	Outpatient visit	During hospitalization	Reference range
Urine analysis	Pus-cells: 1-2/hpf, RBC/Casts: absent	Pus cells: 2-3/hpf, RBC/Casts: absent	Pus cells: 0-5/hpf RBC/Cast: Absent

**Table 3:** Ultrasonography of abdomen during outpatient visit and hospitalization.

Ultrasonography	Outpatient visit	During hospitalization
Ultrasound of abdomen	Urinary debris with Grade I fatty liver.	Left Kidney: Large and swollen with an increased anechoic corticomedullary area, with multiple scattered low-level echoes suggestive of left acute pyelonephritis. Right Kidney: Normal

Two days after the onset of symptoms (Day 3), she presented to the Emergency Medicine Department with intermittent high-grade fever with chills and rigor, unbearable pain in the left flank (costovertebral angle), and dysuria. During examination, she was conscious, oriented, and febrile (100.2^0^F). She had a blood pressure of 86/40mmHg, a pulse rate of 112/minute (low volume), and SpO_2_ of 98% in room air. General examinations were the same without any rashes and ulcers. She had the same systemic examination as before. The only significant examination was left costovertebral angle tenderness without any other system abnormalities. The random blood sugar was 156mg/dL. Initial arterial blood gas analysis showed lactate of 6mg/dL without any other abnormality. Initial electrocardiographically showed sinus tachycardia and the radiograph of the erect abdomen was normal. Two units of 500ml normal saline were rushed and noradrenaline infusion was initiated with a normal saline of 60ml/hour. Foley’s catheterization was done. She was admitted to the High-Dependence Unit (HDU) under the Department of General Medicine. Her vitals and urine output were closely monitored. Complete blood count revealed Hemoglobin: 11.0mg/dL, TLC: 12300 (neutrophilic leukocytosis with toxic granules), ESR: 78mm/hr, RBS: 178mg/dL, serum urea: 156mg/dL, creatinine: 2.1mg/dL, total protein: 8.6mg/dL, and Albumin: 4.5mg/dL (Table 1). Repeat urine analysis showed 4-5 pus cells/hpf, without any reference blood cells and casts. Bedside ultrasonography revealed a large and swollen left kidney with an increased anechoic corticomedullary area, with multiple scattered low-level echoes suggestive of left acute pyelonephritis without any obstructive features with a normal right kidney (Table 3). A provisional diagnosis of type 2 diabetes mellitus with left acute pyelonephritis with septic shock was kept and injection piperacillin tazobactum was started with a modified renal dose in place of tablet cefixime, and injection hydrocortisone 200mg stat dose was given intravenously followed by 50mg intravenous six hourly was continued in view of septic shock and other supportive treatments were continued. 

**Figure 1:**
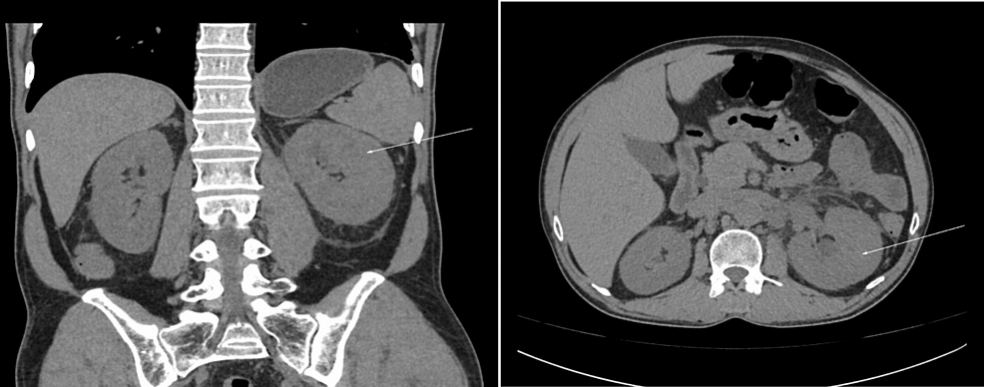
A bulky and globular left kidney with significant perinephric fat stranding with lack of parenchymal gas and thickened left conal fascia with minimal surrounding haziness, likely left acute pyelonephritis (white arrows).

On the next day (Day 4), the urine and blood culture sensitivity report showed no growth, but the serum procalcitonin was 98 ng/ml. She was febrile intermittently, which was readily controlled by paracetamol. The ionotrope requirement increased and the urine output was 700ml/day. Her blood sugar was adequately controlled with regular Insulin preparations. The kidney function test was repeated in the evening and revealed serum urea: 211mg/dL and creatinine: 2.8mg/dL. Her HBsAg, Anti HCV, and human immunodeficiency Virus (I and II) were negative. Non-contrast computerized tomography was done to rule out any obstructive etiology, which showed a bulky and globular left kidney with significant perinephric fat stranding with a lack of parenchymal gas and thickened left conal fascia with minimal surrounding haziness, indicative of left acute pyelonephritis (Figure 1). History, examination, urine analysis, and radiological evidence supported a diagnosis of left acute pyelonephritis. Meticulous history revealed she had a history of a picnic party at a nearby waterfall area 14 days before. The skin surface examination was repeated and a small Escher of 4mm x 4mm was noted in the perianal region. The rapid diagnostic test for malaria was negative. IgM ELISA (enzyme-linked Immunosorbent assay) for dengue, scrub typhus, and Leptospira was advised. 

On the next day (Day 5), she had a recurrent high-grade fever, for which cold sponging and paracetamol infusions were given. Her reports showed IgM for scrub typhus was positive (titer 1: 3200), IgM for dengue and Leptospira was negative, and sterile blood culture (Table 4). Injection doxycycline 100mg intravenous twice daily was added to the treatment. The noradrenaline requirement was the same as before and urine output was also 750ml/day. 

On the 6th day and 7th day, the patient was relatively stable with the above medications. She had no episodes of fever. Her ionotrope requirement decreased and her urine output improved to 1200ml/day. Her repeated complete blood count, and kidney function test showed Hb: 10.8%, TLC: 9900 (neutrophilic), urea: 98mg/dL, and Creatinine: 1.7mg/dL (Table 1). 

Till Day 8 and Day 9, she improved significantly. Her ionotrope support was removed, urine output improved to 1700ml/day and the blood report showed procalcitonin of 67ng/ml. Her intravenous fluid support was reduced. 

Blood routine investigations were repeated on Day 10 which showed hemoglobin: 10.8mg/dL, TLC: 7300 (neutrophilic), RBS: 165mg/dL, serum urea: 21mg/dL, creatinine: 0.9mg/dL, total protein: 8.2mg/dL, albumin: 4.1mg/dL, and procalcitonin: 10ng/ml (Table 1). Her Foley’s catheter was removed. Injection piperacillin tazobactum and injection hydrocortisone were stopped and she was shifted to the general ward for further observation. 

Two days after (Day 12), her blood spouting investigations were repeated and it showed decreased TLC (6800, with 67% neutrophil), urea: 18mg/dL, and creatinine: 0.8mg/dL. Her serum procalcitonin fell to <0.5mg/dL (Table 1). 

On the next day (Day 13), she had no fresh complaints. Her vitals were stable and, her urine output was adequate. She was discharged with tablet doxycycline 100mg one tablet twice daily for five more days, and an injection regular insulin was advised for follow-up in the General Medicine outpatient department (OPD) after one week. Relevant Investigations are mentioned in Tables 1-4.

**Table 4:** Microbiological investigations for various infections. N/A: Not applicable.

Infection Panel	Result	Reference range
RDT for Malaria (Pv/Pf)	Negative (card test)	N/A (Card test)
IgM for Scrub typhus	Positive (1:3200)	>1:400
IgM For Dengue	Negative	N/A (Card test)
IgM for Leptospira	Negative (N/A)	>1:400
RK-39 antigen	Negative (N/A)	>1:128
Urine for AFB (3 samples)	Negative	N/A (Microscopy)

In her follow-up OPD visit, she had no other complaints, and her vitals were blood pressure: 116/68 mmHg, pulse rate: 88/minute, respiratory rate: 18/minute, and RBS: 188mg/dL. Ultrasonography of bilateral kidneys was repeated for academic interest and revealed the normal study. Injection regular insulin was stopped and converted to tablet glimepiride 1mg one tablet once daily and tablet metformin 500mg one tablet once daily. She was sent back home with the advice of strict blood sugar control.

## Discussion

Rickettsial infections are commonly found in Southeast Asia, Northern Australia, and islands of the Western Pacific and Indian Ocean with a high rate of morbidity and mortality. *Orientia tsutsugamushi* differs from other rickettsial diseases in both genetics and cell wall composition. It is maintained by trans-ovarian transmission by trombiculid mites. Environmental exposure and travel history are important while suspecting a rickettsial disease. Scrub typhus can present only with fever with spontaneous resolution of the symptoms. However, manifestations of the disease can be fever, rashes, eschar, pneumonitis, meningoencephalitis, progressive hypotension, and multi-organ dysfunction. Renal involvement in scrub typhus is a significant aspect of the disease progression leading to various clinical manifestations and complications. AKI is a common feature of this disease [5]. The pathogenesis of renal involvement in scrub typhus is multifactorial, involving direct invasion of the kidney by the bacterium, immune-mediated mechanisms, and systemic effects of the infection [6]. They may present with proteinuria, hematuria, and elevated urea and creatinine indicating renal dysfunction. The mechanisms postulated for renal involvement include typhus-related vasculitis, tubular interstitial proliferation, and tubular necrosis [7]. There are fewer case reports showing acute pyelonephritis in patients with scrub typhus, which is rarely found [8,9]. 

In the current case scenario, the patient was initially evaluated in the OPD, where inadequate information was collected, and started with normal blood routine examination, urine analysis, and radiological investigations. After she was admitted to the HDU, even after proper history taking, thorough examination, and adequate investigations, the causative organism of the acute pyelonephritis was unknown. As a tropical country, India caters to a high number of scrub typhus cases and a history of picnic parties, which raised suspicion for investigation. 

An Escher in the perianal region and the positive serology of IgM against scrub typhus antigen confirmed the diagnosis. CT imaging showed a likely diagnosis of left acute pyelonephritis. Injection doxycycline was given intravenously from Day 5 to Day 12. During this period, the patient improved drastically and was discharged with tablet doxycycline. After treatment with the same, her ultrasound findings were normal in the last OPD visit. After two days of initiation of intravenous doxycycline, her fever subsided completely, ionotrope requirement was abolished, urine output improved, and blood investigations showed drastic improvement which favors the diagnosis of scrub typhus-related acute pyelonephritis with septic shock rather than any other acute bacterial sepsis. 

## Conclusions

Urinary tract infections were one of the common diagnoses of acute febrile illness. Common organisms were readily treated with conventional antibiotics and other supportive treatments. As a tropical country, atypical organisms like Orientia tsutsugamushi causing sterile urine culture should be kept in mind. If almost all tertiary care hospitals are equipped with IgM serology against Scrub typhus and with appropriate treatment in time, it can avert life-threatening complications. There should be healthcare awareness and community awareness of the disease transmission, suspicion, pathogenesis, progression, and preventive measures for better management of the infection.
